# *Neisseria gonorrhoeae* among suspects of sexually transmitted infection in Gambella hospital, Ethiopia: risk factors and drug resistance

**DOI:** 10.1186/s13104-016-2247-4

**Published:** 2016-09-13

**Authors:** Seada Ali, Tsegaye Sewunet, Zewdineh Sahlemariam, Gebre Kibru

**Affiliations:** 1 Gambella Teachers and Health Science College, Gambella, Ethiopia; 2 Department of Medical Laboratory Sciences and Pathology, Jimma University, P. O. Box 378, Jimma, Ethiopia

## Abstract

**Background:**

*Neisseria gonorrhoeae* is a bacterium responsible for one of the classic sexually transmitted infection (STI) gonorrhea. Antibiotic resistant strains are emerging at alarming rate. Multiple sexual partners, unsafe sex and substance use habits are the main host related risk factors for acquiring the infection. Thus, this study aimed at determining the magnitude, its determinants and antimicrobial resistance profile of *N. gonorrhoeae* in a place where there is risk related cultural practices and relatively high HIV prevalence.

**Methods:**

A cross-sectional study was conducted on 186 STI suspected patients seen in Gambella hospital from March to July 2015. Data on socio-demographic characteristics and associated risk factors was collected using pre-designed questionnaire. Urethral or endo-cervical swabs were collected aseptically by trained nurses. Then, samples were transported to laboratory and processed within 15 min following standard microbiological culture techniques. Antimicrobial susceptibility test was performed by using Kirby-Bauer disk diffusion method. Data entry, transforming and analysis was done using SPSS version 20.

**Results:**

In this study 11.3 % of the STI suspected patients were confirmed to have *N. gonorrhoeae.* The rate of infection in males was four times higher than in females accounting 16.0 and 5.0 % respectively (p = 0.049). It was also higher (18.9 %) in 20–24 years age group (p = 0.439). Alcohol intake (p = 0.013), less frequent condom use (p = 0.031), and multiple sex partners (p = 0.024) were associated with increased odds of infection. All *N. gonorrhoeae* isolates were susceptible to ceftriaxone and cefoxitin but all were resistant to penicillin and tetracycline. Alarmingly, 28.6 % of the isolates were resistant to ciprofloxacin.

**Conclusions:**

The proportion of urogenital symptoms attributable to *N. gonorrhoeae* was high (11 %), with highest prevalence among males and young adults. Hence, prevention efforts should consider behavioral risk reduction. Ceftriaxone and cefoxitin can be considered as excellent first-line treatment options. However, alarming rate of resistance to ciprofloxacin challenges the current use of this antibiotic in the syndromic management package of gonococcal infections. Thus, laboratory based diagnosis and treatment system is need.

## Background

*Neisseria gonorrhoeae* is a gram negative coffee-bean shaped intracellular diplococcus bacterium responsible for gonorrhea which is one of the classical sexually transmitted infections (STIs) [1]. Gonorrhea can also be transmitted from mother to child during delivery and cause infection of the eye of the newborn [1–3]. Genital tract gonorrhea gives rise to well recognized complications such as pelvic inflammatory disease with possible sequelae including infertility, ectopic pregnancy, fetal wastage, neonatal ophthalmia and disseminated gonococcal (GC) infections. People with gonorrhea infection have increased risk of HIV acquisition and transmission [2, 4, 5].

Antibiotic resistant *N. gonorrhoeae* is emerging at an alarming rate in many parts of the world, especially in developing countries. As a result, inexpensive treatment regimens of gonorrhea in those countries have been rendered ineffective while efficacious ones are often unaffordable [3, 6, 7]. After years of easy susceptibility of *N. gonorrhoeae* to penicillin and other antibiotics, there is a worrying trend of antimicrobial resistance to the commonly prescribed antibiotics such as quinolones and cephalosporins. Although, the Center for Disease Control (CDC) recommends a combination therapy such as, ceftriaxone plus either azithromycin or doxycycline as first-line treatment for gonorrhea, it also noted that as a result of high drug resistant ability of gonococci cephalosporin resistance, especially ceftriaxone resistance, would greatly limit treatment options and cripple gonorrhea control efforts [8].

In 2008 the World Health Organization (WHO) estimated that about 106.1 million new cases of GC infections occurred globally and about 21.1 million in Africa, making it the second most common sexually transmitted bacterial infection worldwide [5]. Moreover, the global prevalence of *N. gonorrhoeae* in adults between the ages of 15 and 49 years was estimated to be 36.4 million in 2008. Meanwhile in Africa the prevalence in these age groups was estimated to be 8.2 million [5]. Some of the recent studies in Africa showed that the prevalence of the disease in STI suspected patients ranges from 2.7 to 8.2 % in various target groups [6, 7]. Up to 17.7 % gonorrhea prevalence was also detected among anti-retroviral treatment (ART) attendees [9]. As indicated, in sexually transmitted diseases treatment guideline, multiple sexual partners, sexually active age, unsafe sex practice, lower socio-economic status, urban residence and substance use are among the list of host related risk factors for acquiring the infection [10].

The few available reports on prevalence of *N. gonorrhoeae* in Ethiopia showed significant variation in its distribution among the regions. Due to unavailability of culture-facility in health institutions in the country, the treatment of gonorrhea is almost empirical and antibiotics are generally given without considering sensitivity report. Recently in any health facilities the STI case are routinely managed by syndromic approach [11, 12] where patients suspected of *N. gonorrhoeae* infection are treated empirically by giving antibiotics recommended in the package. On the other hand, Gambella is one of the regions where polygamy and levirate marriages are very common [13] and male circumcision is rare [14]. Such practices are expected to be risks for STI and HIV acquisition. This might explain why the prevalence of HIV in the region ranks the highest (6.5 %) in Ethiopia [15]. Despite this risk related cultural practices and relative high HIV prevalence in the region, there is no adequate information about the magnitude, its determinants and antimicrobial resistance status of *N. gonorrhoeae*.

## Methods

### Study area and design

A cross sectional study was conducted from March to July 2015 on STI suspected patients attending the outpatient department (OPD) of Gambella hospital, Gambella regional state, Ethiopia. The total annual patient flow of the hospital in 2014 was 244,656 of which 580 were STI cases. Syndromic approach is often used to manage STI cases in Gambella hospital and in all other health care facilities in the region. In this approach, patients suspected of *N. gonorrhoeae* infection, are managed by giving antibiotics such as ciprofloxacin 500 mg tablet by mouth stat (commonly), or spectinomycin 2 g intramuscular (IM) stat (occasionally) or ceftriaxone 250 mg IM as a sing dose (for recurrent infection) in combination with other antibiotics whenever required [11, 12].

The minimum representative sample size was determined using single population proportion formula by taking 8.2 % prevalence rate of *N. gonorrhoeae* documented in the same country [7] is with margin of error 4.1 at 5 % level of significance. All STI suspected patients whose age ≥15 years and had no history of antibiotic treatment in the preceding 2 weeks were included in the study. Written consent was obtained from study participant patients prior to data collection. Then, information on sociodemographic and other variables of interest was collected by trained data collectors using pre-designed questioners.

### Sample collection, and processing

Urethral or endo-cervical swabs (two swabs from each individual) were collected aseptically following standard procedures [16] by trained nurses. Urethral swabs from men were collected by gently massaging the urethra down wards from above using sterile cotton wool swabs. For those who had no noticeable pus the swabs were inserted approximately 2 cm into their urethra and rotated gently before withdrawing. In females, the cervical swabs were collected from the endocervical canal using well disinfected vaginal speculum. Then, the swabs were immediately put in Amies transport media (Oxoid limited, Basingstoke, RG24 8PW, UK), transported to the Gambella Regional Laboratory and processed within 15 min of collection [16].

### Bacteria identification

Of these two swabs, one was used for Gram stain and the other was inoculated directly into the modified Thayer Martin medium (MTM) and incubated at 37 °C for 24–48 h in a moist atmosphere enriched with 5–10 % CO_2_ (i.e. by putting soaked gauze inside the candle jar). Positive culture was identified by its characteristic appearance on the media (i.e. small raised, grey shiny colonies after overnight incubation on MTM). Suspected single colony from MTM was taken and subcultured on a GC-chocolate agar with 1 % Vitox supplement and incubated for overnight. Confirmation of *N. gonorrhoeae* isolates was done by Gram-stain, Biochemical tests (including oxidase, superoxol & carbohydrate utilization tests) [16] and using Analytical Profile Index for Identification of *Neisseria* and *Hemophilia* (API NH) identification kit strips (BioMerieux, France). Isolates that are oxidase positive, superoxol positive and fermenting only glucose were considered as *N. gonorrhoeae* [17, 18].

### Antimicrobial susceptibility testing

Antimicrobial susceptibility testing was performed for all isolates according to the criteria of Clinical and Laboratory Standard Institute (CLSI) by the Kirby-Bauer disk diffusion method [19]. Bacterial suspensions with turbidity standard equivalent to McFarland 0.5 were swabbed evenly on GC-chocolate agar with 1 % Vitox supplement. A set of six antibiotic discs (Oxoid Ltd., Basingstoke, Hampshire, England) with the following concentrations: tetracycline 30 µg, penicillin 10 Iu, ciprofloxacin 5 µg, spectinomycin 100 µg, cefoxitin 30 µg and ceftriaxone 30 µg were placed on the surface of the plate. Then, the plates were incubated at 37 °C in candle jar, generating 5–10 % CO_2,_ for 20–24 h. Zone of inhibition diameters in mm were measured and interpreted as sensitive, intermediate and resistant according to the principles established by CLSI [19]. In order to monitor quality (potency) of disks, a standard strain of *N. gonorrhoeae* American Type Culture Collection (ATCC) 49226 was tested at regular interval. The tested antibiotics were selected based on the national list of medicines by Food, Medicine and HealthCare Administration and Control Authority (FMHACA) Ethiopia in 2010 to treat infections, Syndromic management package for the management of sexually transmitted infections, Federal democratic repuplic of Ethiopia [11, 12] and prescription frequencies.

### Data analysis

Data entry, transforming and analysis was done using SPSS version 20. Frequency of variables was determined and descriptive findings were presented using tables and graphs. Crude odds ratio (COR) and adjusted odds ratio (AOR) with 95 % confidence interval (CI) were calculated. p value was calculated to identify statistical significance. Binary logistic regression was used to assess the associations between dependent (*N. gonorrhoeae* infection) and independent (demographic and behavioral) variables. Variables with p value less than 0.25 were taken as candidates to enter multiple logistic regression model and variables with p < 0.05 were reported with 95 % CI and AOR as determinant factor for *N. gonorrhoeae* infection.

## Results

### Socio-demographic characteristics of the study participant patients

**Fig. 1:**
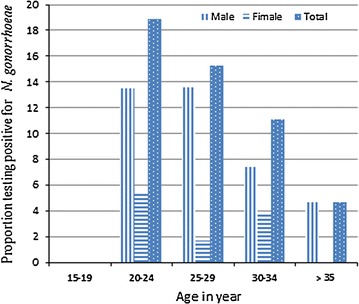
Proportion of *N. gonorrhoeae* by age group and sex of STI suspected patients (n = 186) in Gambella hospital, Ethiopia, (March–July 2015)

During the study period a total of 189 STI suspected patients were seen in Gambella hospital outpatient department (OPD) of which 186 of them gave their consent to participate. The sex and age profile of participants showed that 106 (57 %) were males and their age ranged from 15 to 65 years the mean age being 28.9 ± 8.3 years. Out of 186 participants whose urethral or endocervical swabs were investigated, 21 (11.3 %) were confirmed to have gonococcal (GC) infection. The highest frequency of GC infection occurred among 20–20 year-olds (19 %) as compared to 15–19 year-olds in which no infection was seen (0 %) and older age groups (Fig. 1). However, the difference was not statistically significant (Table 1).

**Table 1:** Distribution of *N. gonorrhoeae* infection in relation to socio-demographic characteristics of STI suspected patients (n = 186) seen in Gambella hospital, Ethiopia (March–July 2015)

Socio-demographic characteristics	*N. gonorrhoeae* infection		COR (95% CI)	p value	AOR (95% CI)	p value
	Positive (n = 21) n (%)	Negative (n = 165) n (%)				
*Gender*						
Male (106)	17 (16.0)	89 (84.0)	3.63 (1.17, 11.25)	0.026	4.24 (1.01, 17.82)	0.049
Female (80)	4 (5.0)	76 (95.0)	1*		1*	
*Age*						
15–19 (20)	0 (0.0)	20 (100.0)	0.000 (0.000)	0.439		
20–24 (37)	7 (18.9)	30 (81.1)	4.78 (0.93, 24.67)			
25–29 (59	9 (15.3)	50 (84.7)	3.69 (0.76, 18.04)			
30–34 (27)	3 (11.1)	24 (88.9)	2.56 (0.40, 16.44)			
>35 (43)	2 (4.7)	41(95.3)	1*			
*Residence*						
Urban (134)	12 (9.0)	122 (91.0)	1*	0.112	1*	0.477
Rural (52)	9 (17.3)	43 (82.7)	2.13 (0.84, 5.40)		1.51(0.49, 4.66)	
*Marital status*						
Married (112)	14 (12.5)	98 (87.5)	1*	0.523		
Unmarried (74)	7 (9.5)	67 (90.5)	0.73 (0.28, 1.91)			
*Educational status*						
Illiterate (19)	2(10.5)	17 (89.5)	0.77 (0.16, 0.67)	0.585		
Primary (1–8) (62)	5 (8.1)	57 (91.9)	0.57 (0.195, 1.668)			
S & A (105)	14 (13.3)	91 (86.7)	1*			
*Occupation*						
Employee (80)	12 (15.0)	68 (85.0)	1*	0.978		
House wife (25)	0 (0.0)	25 (100)	0.000 (0.000)			
Farmer (13)	1 (7.7)	12 (92.3)	0.47 (0.06, 0.96)			
Student (39)	5 (12.8)	34 (87.2)	0.83 (0.27, 2.56)			
Merchant (18)	2 (11.1)	16 (88.9)	0.708 (0.14, 3.48)			
Others (11)	1 (9.1)	10 (90.9)	0.57 (0.07, 4.84)			
*AMI (USD)*						
No income (15)	2 (13.3)	13 (86.7)	1.33 (0.26, 6.68)	0.225	10.82 (1.31, 89.63)	0.173
<25 (38)	2 (5.3)	36 (94.7)	0.48 (0.10, 2.27)		2.89 (0.42, 19.86)	
25–50 (27)	6 (22.2)	21 (77.8)	2.47 (0.82, 7.42)		1.78 (0.427, 7.40)	
>50 (106)	11(10.4)	95 (89.6)	1*		1*	

*S & A* secondary and above, *AMI* average monthly income, *1* ^*^ indicator

The prevalence of GC infection in males was much higher than females accounting for 16 and 5 % respectively i.e. the odds of having infection in men was four-times higher than women (p = 0.049, AOR = 4.24, (95 % CI 1.01, 17.82). The prevalence of infection was higher among rural residents (17.3 vs. 9.0 % urban), married person (12.5 vs. 9.5 % unmarried), employees (15 vs. 11.1 % merchants), higher educated (13.3 vs. 10.5 % illiterate and 8.1 % primary school) and those with medium income (22.2 % USD 25–50 vs. 5.3 % USD <25 and 11.4 % USD >50). However, the residential set up and any of those tested sociodemographic variables showed no statistical association with the GC infection (Table 1).

### Substance use and sexual risk behavior of participant patients

The assessment on sexual risk behavior and substance use showed that the rate of GC infection among those who ever use shisha and chew Khat (*Catha edulis*) was 27.3 %, (p = 0.100) and 17.2 %, (p = 0.276) respectively. The rate of infection among cigarette smokers was 38.1 % [(p = 0.015), AOR (95 % CI) = 4.84 (1.35, 17.34)] and those who often drink alcohol was 19.3 % [(p = 0.013), AOR (95 % CI) = 5.53(1.44, 21.24)]. The verified data indicates that cigarette smoking and frequent alcohol drinking habit showed statistically significant association with GC infection (Table 2).

**Table 2:** Distribution *N. gonorrhoeae* infection in relation to substance use, and sexual risk behaviors of STI suspected patients (n = 186) seen at Gambella hospital, Ethiopia (March–July 2015)

Substance use and sexual risk behavior	*N. gonorrhoeae* infection		COR (95 % CI )	p value	AOR (95 % CI)	p value
	Positive	Negative				
	(n = 2) n (%)	(n = 165) n (%)				
*Shisha use*						
Yes (11)	3 (27.3)	8 (72.7)	3.27 (0.80, 13.45)	0.100		
No (175)	18 (10.3)	157 (89.7)	1*			
*Alcohol intake*						
Yes (83)	16 (19.3)	67 (80.7)	4.68 (1.64, 3.39)	0.004	5.53 (1.44, 21.24)	0.013
No (103)	5 (4.9)	98 (95.1)	1*		1*	
*CS*						
Yes (21)	8 (38.1)	13 (61.9)	7.20 (2.53, 20.50)	<0.001	4.84 (1.35, 17.34)	0.015
No (165)	13 (7.9)	152 (92.1)	1*		1*	
*Chewing khat*						
Yes (29)	5 (17.2)	24 (82.8)	1.84 (0.62, 5.48)	0.276		
No (157)	16 (10.2)	141 (89.8)	1*			
*Condom use*						
Yes (39)	2 (5.1)	37 (94.9)	1*	0.188	1*	0.031
No (147)	19 (12.9)	128 (87.1)	2.75 (0.61, 2.34)		7.92 (1.21, 51.90)	
*NSP*						
1–2 (172)	15 (8.7)	157 (91.3)	1*	0.001	1*	0.024
>2 (14)	6 (42.9)	8 (51.1)	7.85 (2.40, 25.64)		6.12 (1.27, 29.86)	

*CS* cigarette smoking, *NS* number of sexual partner, *1* ^*^ indicator

Out of 14 respondents who had multiple sexual partner, 42.9 % were positive for GC infection (p = 0.024) with an AOR = 6.12 and 95 % CI = 1.27, 29.86. The rate of infection among these positive males with multiple sexual partners was fivefold greater than women with the same category i.e. 37.7 % vis-a-vis 7.1 % (data not shown). The proportion of STI due to *N. gonorrhoeae* was 12.9 % among those who reported not using condoms compared to 5.1 % among those who reported condom use (p = 0.188). The odds of having GC infection among those who never use condom was eight times higher than who use regularly [AOR = 7.92 (95 % CI = 1.21, 51.90)] (Table 2).

### Antimicrobial drug resistance pattern

*Neisseria gonorrhoeae* isolates were 100 % susceptible to ceftriaxone and cefoxitin. But, they were 100 % resistant to penicillin and tetracycline. Alarming rate (28.6 %) of resistance was also seen against ciprofloxacin. Moreover, intermediate resistance was seen in 4.8 % of the isolate for spectinomycin and 14.3 % for ciprofloxacin (Table 3).

**Table 3:** Antimicrobial drug resistance patterns of *N. gonorrhoeae* isolates from STI suspected patients at Gambella hospital, Ethiopia (March–July 2015)

Organism	Pattern	Drugs tested No (%)					
		FOX	CRO	SPT	CIP	P	T
*N. gonorrhoeae* (n = 21)	S	21 (100)	21 (100)	20 (95.2)	12 (57.1)	0	0
	I	0	0	1 (4.8)	3 (14.3)	0	0
	R	0	0	0	6 (28.6)	21 (100)	21 (100)

*S* sensitive, *I* intermediate, *R* resistant, *FOX* cefoxitin, *CRO* ceftriaxone, *SPT* spectinomycin, *CIP* ciprofloxacin, *P* penicillin, *T* tetracycline

## Discussion

In this study, the prevalence of *N. gonorrhoeae* among suspected STI patients was 11.3 %, which is higher than 5.1 % reported in Hawassa, Ethiopia [6], 8.2 % in Bahir Dar, Ethiopia [7], 1.2 % in Bangladesh [4] and 8.4 % in Tanzania [20]. This relative increased rate of gonococcal (GC) infections seen in our study might be due to risky cultural practices in the region where there is poly gamy, levirate marriage [13] and rare male circumcision [14] which purporting to show that circumcision reduced the risk of gonorrhea infection [21]. Such practices could be precipitating risk factors for STI and HIV acquisition as the prevalence of HIV in the region is reported to be the highest in Ethiopia [15]. The relative high rate of infection might also goes with the analogy that the presence of other sexually transmitted disease (STD) and HIV increases the risk of acquiring *N. gonorrhoeae* and vice versa [10]. Another factor that contributes for the higher infection rate might be lack of good clinical and diagnostic facilities in the region which intern weakens STI management systems (i.e. strict case identification, counseling and treatment). In contrast to these, the 11.3 % prevalence rate obtained in present study was lower than 17.7 % a report in Jimma, Ethiopia [9], 42 % in Bangladesh [22], 59 % in Uganda [23] and 80 % in Malawi [24]. The variation in prevalence rate in those studies may be due to differences in target population where ART follow up cases in Jimma, commercial sex workers in Bangladesh and patients only with urethral discharge in Malawi were included in their study.

In our study, the odds of GC infection in males was four-times higher than females of the same age group [(P = 0.049), AOR = 4.24, (95 % CI, 1.01, 17.82)]. This might be due to the aforementioned risky cultural practices a in the region [13, 14]. By nature, males are more symptomatic for the infection which may also enhance their health care seeking behavior and more likely appeared in the statistics. Gonococcal infection in this study was also more frequent in 20–24 and 25–29 years of age groups. It is documented that sexually active age groups are at risk of acquiring STIs [10] as they are much more prone to sexual promiscuity and unsafe sex practices.

The bivarate and multivariate analysis on substance use in this study revealed that unlike shisha use and khat chewing practices, alcohol drinking (p = 0.013, AOR = 5.53, 95 % CI = 1.44–21.24) and cigarette smoking (p = 0.015, AOR = 4.84, CI = 1.35–17.34) showed statistical significant association with GC infection. This is due to the fact that alcohol drink and cigarette smoking are cofactors as individuals are more motivated to take risks of unsafe sex while under the influence of alcohol.

Moreover, the logistic regression analysis on sexual risk behavior such as having multiple sexual partner (p = 0.024, AOR = 6.12, 95 % CI = 1.27, 29.86) and condom use habit (p = 0.031, AOR = 7.92, 95 % CI = 1.21–51.90) also showed statistically significant association with the infection. Participants with lack of condom use and multiple sex partners had several times the odds of GC infection. Association of GC infection with two predictor variables (i.e. alcohol drinking and condom use) was documented in similar study on ART attendees in Jimma, Ethiopia [9]. Statistical association of GC infection with study participants having multiple sexual partners was also reported in South Africa [25] and Tanzania [20].

In this study all isolates were 100 % susceptible to ceftriaxone which was analogous to 100 % susceptibility reported in Nepal [26], India [27], Brazil [28], Tanzania [20] and Ethiopia [6]. Our finding was also comparable to 99 % susceptibility towards the same antibiotic documented in Bangladesh [22]. Such effectiveness of ceftriaxone might be associated with limited use and prescription of the drug in the study area because of its high cost. In contrary to our findings, 27.8 % resistant isolates to ceftriaxone was reported in Bahir Dar, north west Ethiopia [7]. However, it is difficult to give possible explanation for such contrasting findings documented in the same country.

The *N. gonorrhoeae* isolates in this study was 100 % susceptible to cefoxitin which is higher than 82 % susceptibility reported in Hawassa, Ethiopia [6]. Similarly, our isolates were 95.2 % susceptible to spectinomycin which is also higher than 82 % susceptibility reported in Hawassa [6]. These two antibiotics remain effective in the area because the drugs are newly introduced so that not used widely and frequently. However, 95.2 % susceptibility to spectinomycin obtained in this study was somehow lower compared to each 100 % susceptibility documented in Bangladesh [22] and Brazil [28].

In this study, *N. gonorrhoeae* isolates showed alarming rate (28.6 %) of resistance against ciprofloxacin which is higher than 18 % resistance to the same drug reported in Ethiopia [6], 20 % in Nepal [26], 21.4 % in Brazil [28] and 23.3 % in Uganda [23]. Such relative high rate of resistance seen in this study might be due to easly availability, indiscriminate and intensive use of this antibiotic in all health facilities in the country. Since ciprofloxacin is included in the syndromic management package, it is often used in combination with doxycycline or azithromycin for infections concomitant with *Chlamydia trachomatis.* It is also widely used both for other STDs and various bacterial infections. The alarming percentage of resistance against ciprofloxacin observed in our study challenges the current use of this drug in the syndromic management package of GC infections. Hence, switching from ciprofloxacin to ceftriaxone was optimal when the prevalence of gonorrhea was >3 % and the prevalence of ciprofloxacin resistance was >5 % as it was suggested by Roy K [31].

In this study, all the isolates showed 100 % resistant to penicillin and tetracycline. Such an extreme resistance against these antibiotics is due to the fact that about 90.5 % of our isolates were tested as penicillinase producers [18]. The 100 % resistance seen in our study was higher than 82 % penicillin resistance reported in Hawasa, Ethiopia [6] 86.6 % in Gonder, Ethiopia [29] and 77 % in Addis Ababa [30]. The variation in rate of penicillin resistance *N. gonorrhoeae* isolates in the same country might be an indication of the overtime period rising in resistance trend of the bacteria to this drug.

## Conclusions

In general, the prevalence of GC infection in the study area was relatively high as compared to findings documented in other regions of the country. Some of the risky cultural practices, substance use and sexual risk behaviors of the individuals showed an association in acquiring the infection. Therefore, behavioral and sexuality education is required for those sexually active populations in that locality as some of the risk factors indicate knowledge gap.

The absence of resistant *N. gonorrhoeae* isolates to ceftriaxone and cefoxitin in our study makes these antibiotic excellent first-line treatment options. However, the alarming percentage of resistance against ciprofloxacin could significantly challenge the current use of this antibiotic in the syndromic management package of GC infections. Therefore, laboratory based diagnosis and antimicrobial susceptibility test systems need to be in place.

## Abbreviations

HIV: human immunodeficiency virus
GC: gonococcal
STI: sexually transmitted infections
CDC: Center for Disease Control
WHO: World Health Organization
ART: anti-retroviral treatment
MTM: Thayer Martin medium
CLSI: Clinical and Laboratory Standard Institute
COR: crude odds ratio
AOR: adjusted odds ratio
CI: confidence interval
